# First Report of Native Parasitoids of Fall Armyworm *Spodoptera frugiperda* Smith (Lepidoptera: Noctuidae) in Mozambique

**DOI:** 10.3390/insects11090615

**Published:** 2020-09-08

**Authors:** Albasini Caniço, António Mexia, Luisa Santos

**Affiliations:** 1 LEAF-Linking Landscape, Environment, Agriculture and Food- School of Agriculture—University of Lisbon, Tapada da Ajuda, 1349-017 Lisbon, Portugal; amexia@isa.ulisboa.pt; 2 Division of Agriculture—The Polytechnic of Manica (ISPM), District of Vanduzi, Matsinho 2200, Mozambique; 3 Postgraduate Program Science for Development (PGCD), Gulbenkian Institute of Science, Rua da Quinta Grande 6, 2780-156 Oeiras, Portugal; 4 Department of Plant Protection-Faculty of Agronomy and Forestry Engineering, Eduardo Mondlane University, P.O. Box 257, Maputo 1102, Mozambique; luisasantos47@gmail.com

**Keywords:** fall armyworm, invasive species, parasitoids, biological control, Mozambique

## Abstract

**Simple Summary:**

In 2016, a highly destructive insect pest with origin in the Americas was detected in Africa. The pest is known to feed primarily on maize which is a staple food in the continent. Since then, farmers have been using chemical insecticides to control the pest. Chemical insecticides are expensive and harmful to the environment. In this article, the authors Albasini Caniço, António Mexia, and Luisa Santos discuss the possibility of application of an alternative method of control known to be environmentally friendly and economically sustainable in the long term. The method, known as “biological control”, can be easily implemented by farmers, and has the potential to reduce the population of the insect pest and production costs, and bring long term benefits to the environment.

**Abstract:**

The alien invasive insect pest *Spodoptera frugiperda* Smith (Lepidoptera: Noctuidae), commonly referred to as fall armyworm (FAW), is causing significant losses to maize production in Africa since its detection in 2016. As an emergency response, governments in several countries distributed and/or promoted massive use of synthetic insecticides among smallholder farmers to fight FAW. The inappropriate use of synthetic insecticides by non-trained and ill-equipped farmers raises environmental and health concerns. This study aimed to assess the occurrence of native parasitoids of FAW, their parasitism rates, and relative abundance in the central province of Manica, Mozambique. A field collection of FAW egg masses and larvae was conducted from May to August 2019 (dry season of the 2018/2019 cropping season) and in December 2019 and January 2020 (rainy season of 2019/2020 cropping season). A total of 101 egg masses and 1444 larvae of FAW were collected from infested fields. Five larval parasitoids were recorded, but no egg parasitism was observed. *Coccygidium luteum* Brullé (Hymenoptera: Braconidae) and *Drino quadrizonula* Thomson (Diptera: Tachinidae) were the primary parasitoids. Maximum parasitism of 23.68% and 8.86% and relative abundance of 100 and 96.3 were recorded for *C. luteum* and *D. quadrizonula*, respectively. Total parasitism by different parasitoid species was at 9.49%. Cultural practices favoring the action of these parasitoids should be advocated.

## 1. Introduction

The fall armyworm *Spodoptera frugiperda* Smith (Lepidoptera: Noctuidae) is an alien polyphagous insect pest originating from the Americas, where it has more than 350 different host plants including both crop and non-crop species [1]. Despite its ability to survive in different host plants, fall armyworm (FAW) is known to have a high preference for maize [2,3]. In Africa, FAW was first reported in West and Central Africa in 2016 [4] and rapidly spread to the rest of the continent with devastating consequences on maize production [5]. Initially confused with stem borers by agricultural extension officers, the occurrence of FAW in Mozambique was confirmed in early 2017 by the Ministry of Agriculture and Food Security [6]. In 2018, FAW was also reported in Asia [7]. The rapid spread of FAW is attributed mainly to its migratory potential [8] and high dispersal capacity [9].

Alien invasive species are known to disrupt the natural balance in newly invaded ecosystems, creating severe problems [10]. This is the case of FAW, which threatens food security in Sub-Saharan Africa, where maize is a staple food [11,12,13,14]. In Mozambique, for example, 21–90% of the households depend on maize for daily subsistence [15].

Before the arrival of FAW in Africa, it was estimated that more than 97% of smallholder farmers did not use any chemicals for pest management on maize production. However, that scenario changed immediately after the detection of FAW, because governments in various countries started distributing and/or promoting the use of synthetic insecticides as an emergency response [9,16,17]. As farmers did not receive accurate information from agricultural services on which insecticides to apply and how and when to apply, they mostly decided on their own, leading to indiscriminate use of insecticides both in terms of type and dose of application.

The continuous and arbitrary use of synthetic insecticides by farmers with no adequate training on pesticides management and application may induce the development of resistance of FAW to these insecticides as was the case in Puerto Rico and Mexico [18]. Additionally, it contributes to environmental pollution and the killing of beneficial insects. It also raises public health concerns as most farmers do not use adequate application and protection equipment when spraying their fields. Furthermore, the effective application of insecticides requires some knowledge of the biology and ecology of the pest being targeted, and that was not the case for FAW in Africa [19]. For these reasons, the use of insecticides should not be viewed as a stand-alone technique, but as a component of an Integrated Pest Management (IPM) scheme [20]. In the IPM approach, natural enemies can play an important role in the management of FAW [21].

In Mozambique, since its detection, FAW has been mainly managed using chemical insecticides, and no information is available regarding the potential for biological control through native parasitoids. Biological control has the potential to bring economic, health, and environmental benefits in the long term.

In its native habitat, FAW is attacked by several natural enemies including parasitoids and entomopathogenic fungi [22,23,24,25] which target different development stages [21,26,27,28] causing significant mortality on its population [29,30,31]. Around 17 different parasitoid species of FAW are known in its native range [29]. In Mexico, a complex of larval parasitoids belonging to Ichneumonidae, Braconidae, Eulophidae, and Tachinidae families were reported [2,23,24,26,31]. In Florida, larval parasitoids of FAW were also reported [27,28]. In Honduras, the main natural enemies of FAW are also larval parasitoids [30]. In Brazil, FAW eggs are primarily parasitized by *Trichogramma* spp. (Hymenoptera: Trichogrammatidae) [21].

In Africa, several parasitoids attacking different stages of FAW were reported in various countries. Six larval parasitoids were collected in Ethiopia, four larval parasitoids and one egg parasitoid were collected in Kenya, and four larval parasitoids were collected in Tanzania [32]. *Telenomus remus* Nixon (Hymenoptera: Scelionidae), an important egg parasitoid of *Spodoptera* spp. (Lepidoptera: Noctuidae) was found parasitizing eggs of FAW in South Africa, Côte d’Ivoire, Niger, Benin and Kenya [33] and also in Ghana [34]. A complex of egg, egg–larval, larval, and larval–pupal parasitoids of FAW including *T. remus*, *Trichogramma* sp., *Chelonus bifoveolatos* Szépligeti (Hymenoptera: Braconidae), *Coccygidium luteum* (Brullé) (Hymenoptera: Braconidae), *Cotesia icipe* Fernandez-Triana and Fiaboe (Hymenoptera: Braconidae), *Meteoridea* cf. *testacea* (Granger) (Hymenoptera: Braconidae), *Charops* sp. (Hymenoptera: Ichneumonidae), *Metopius discolor* Tosquinet (Hymenoptera: Ichneumonidae), *Pristomerus pallidus* (Kriechbaumer) (Hymenoptera: Ichneumonidae), and *Drino quadrizonula* (Thomson) (Diptera: Tachinidae) were reported in Ghana and Benin [34]. Different parasitoid species including *Bracon* sp. (Hymenoptera: Braconidae), *Anatrichus erinaceus* Loew (Diptera: Chloropidae), and an unidentified tachinid were also reported in Ghana [35]. This study aimed to assess the occurrence of native parasitoids of FAW, their parasitism rates, and relative abundance for potential application in biological control programs.

## 2. Materials and Methods

### 2.1. Description of the Study Area

**Figure 1:**
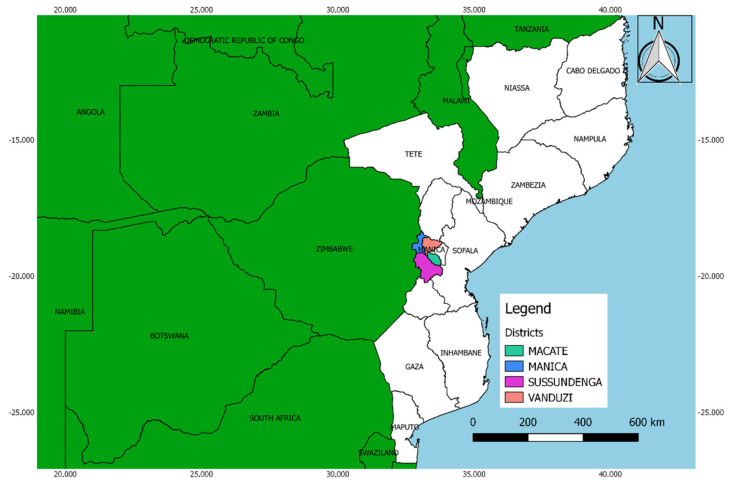
Sampling locations in Mozambique.

This study was carried out in the districts of Macate (19°24′50.9″ S and 33°30′54.6″ E), Manica (18°56′13.2″ S and 32°52′33.6″ E), Sussundenga (19°24′39.0″ S and 33°16′33.0″ E), and Vanduzi (18°57′09.4″ S and 33°15′51.6″ E) in the central province of Manica, Mozambique (Figure 1). According to MASA [15], the area of the survey belongs to the agro-ecological region (AER) number 4, which is characterized by the extensive occurrence of ferralsols and lithosols with an annual mean temperature around 24 °C and annual mean precipitation ranging between 800 and 1000 mm. During the dry season, maize is cultivated mainly in areas with irrigation systems or in valleys and riverbanks with sufficient soil moisture and in small plots (less than 1 ha), under different cropping systems and mainly for family consumption. In general, no fertilizers and chemicals are used for maize production at smallholder farmers’ level. Maize is usually intercropped with roots and tubers, legumes and cucurbits. Vegetables such as tomatoes, cabbage, and kale are typically grown in monocrop systems occasionally adjacent to maize fields. Surrounding environment is mainly dominated by fruit trees like avocado, banana, mango, litchi, and gramineous plants.

### 2.2. Field Collection of FAW Egg Masses and Larvae

A field collection of FAW egg masses and larvae was carried out from May to August 2019 (dry season of the 2018/2019 cropping season) and in December 2019 and January 2020 (rainy season of the 2019/2020 cropping season). A total of 622 maize fields were surveyed including 25 and 131 fields in Macate, 29 and 137 fields in Manica, 27 and 141 fields in Sussundenga, and 59 and 73 fields in Vanduzi in the dry and rainy seasons, respectively. Districts were selected based on their potential for maize production combined with the reported occurrence of FAW. Each field was visited once during the study period. Fields were selected through snowball sampling techniques. Only fields with at least 200 plants were selected. Based on the illustration of maize growth stages by Beckingham [36], only maize fields in which plants were in stages 1–5 were sampled as described: (stage 1): five leaves fully emerged; (stage 2): eight leaves fully emerged; (stage 3): 12 leaves; (stage 4): 16 leaves; (stage 5): Tasseling/Silking. In each field, plants with visible FAW attack symptoms were intentionally selected and checked for the presence of FAW egg masses and larvae. Stalks, whorls, and both upper and lower surfaces of plant leaves were inspected. The number of plants inspected and the number of FAW egg masses and larvae collected varied among fields, as a consequence of the number of damaged/infested plants per field. FAW egg masses and different larval stages were collected from infested maize plants together with a piece of a fresh leaf so that larvae could continue feeding. Egg masses were temporarily placed in bulk into 50 mL transparent plastic vials. FAW larvae were placed in a transparent plastic bowl covered with a mesh and transferred to the entomology laboratory at Instituto Superior Politécnico de Manica. Given that the pupal stage of FAW occurs typically in the soil, this stage was deliberately excluded from the survey. Sprayed fields were also excluded from the survey.

### 2.3. Laboratory Handling of Field-Collected Material

In the laboratory, FAW egg masses and larvae were counted and separated per district and date of collection. Individual egg masses were transferred to 2.5 mL Eppendorf tubes and covered with cotton wool. Larvae were transferred to individual plastic vials with small holes in the lid to allow ventilation. Larvae were fed with clean and non-treated pieces of fresh maize leaves grown in a greenhouse. Both egg masses and larvae were reared at an ambient temperature varying between 26 and 30 °C. Every 48 h, feces of feeding larvae were removed from the vials and vials were cleaned with cotton wool before adding new pieces of fresh maize leaves. Daily, egg masses and larvae were checked for parasitism. After the emergence of parasitoids, dead FAW larvae were removed from the vials. Unparasitized FAW larvae were allowed to reach the adult stage and used for a separate study. FAW larvae hatching from unparasitized egg masses were also used in a separate study. The number of individuals of each parasitoid species emerged from parasitized larvae was recorded. The behavior (endo/ectoparasitic) and trait (solitary/gregarious) of each parasitoid species were recorded. The behavior and trait of each parasitoid were determined based on laboratory observations. Emerged adult parasitoids were preserved in 70% alcohol and frozen at −27 °C. The parasitoids were sent for morphological identification to CABI Switzerland, which hosts a collection of parasitoids attacking FAW in Africa and is presently preparing an identification key and descriptions for all recorded species (M. Kenis, personal communication). Voucher specimens are preserved at CABI Switzerland.

### 2.4. Relative Abundance of Parasitoids

The relative abundance of each parasitoid species (RA) was determined by dividing the number of individuals of a given parasitoid species (ni) by the total number of individuals of all parasitoid species (N) and converted to percent values (Equation (1)).

(1) $$\mathrm{RA}=\frac{\mathrm{ni}}{N}*100\%$$

### 2.5. Parasitism Rates

The parasitism rate of each parasitoid species (Pp) was determined by dividing the number of parasitized larvae (Lp) by the number of collected larvae (TL) and converted to percent values (Equation (2)). Gregarious parasitoids emerging from a single larva were considered as being only one. Parasitism rate of the egg masses was not calculated as none were parasitized.

(2) $$\mathrm{Pp}=\frac{\mathrm{Lp}}{\mathrm{TL}}*100\%$$

### 2.6. Survival of Parasitoids

Larvae of different parasitoids emerging from FAW larvae were counted and monitored until the emergence of adult individuals. Larvae of parasitoids were reared at ambient temperature described in Section 2.3. The number of individuals of different parasitoids which died at larval/pupal stage and those reaching the adult stage was recorded (Table S1). Not a single FAW larvae or pupa was dissected to search for dead parasitoids. The survival rates of different larval parasitoids (SR) was determined by dividing the number of individuals reaching the adult stage (Pa) by the number of individuals emerging from field collect FAW larvae (Pe) and converted to percent values (Equation (3)).

(3) $$\mathrm{SR}=\frac{\mathrm{Pa}}{\mathrm{Pe}}*100\%$$

### 2.7. Relative Contribution to Total Parasitism

The relative contribution of each parasitoid species to total parasitism (RP) was determined by dividing the total number of FAW larvae parasitized by each parasitoids species in both seasons (PS), by the total number of FAW larvae collected in both seasons (LS) and converted to percent values (Equation (4))

(4) $$\mathrm{RP}=\frac{\mathrm{PS}}{\mathrm{LS}}*100\%$$

## 3. Results

### 3.1. Distribution of FAW Parasitoids

A total of 101 FAW egg masses were collected, but no egg parasitoids were detected. Five different larval parasitoids were collected from 1444 FAW larvae. Recorded parasitoids were distributed in three different families: *C. luteum*, *Charops* sp., *M.* cf. *discolor*, Unidentified (Diptera: Tachinidae), and *D. quadrizonula*. *M*. cf. *discolor* and the unidentified tachinid could not be identified with certainty because only one male specimen was collected. Parasitoids were found to be differently distributed among districts and between seasons. Three parasitoid species were recorded in Macate, three in Manica, four in Sussundenga, and two in Vanduzi. Out of all five parasitoid species, *C. luteum* was the only parasitoid recorded in all districts in both seasons (Table 1).

### 3.2. Survival of Parasitoid Species

Table 2 shows the survival rates of different parasitoid species emerging from field-collected FAW larvae. The two most common species *C. luteum* and *D. quadrizonula* reached maximum survival rates of 52.63% and 88.44% respectively. The numbers shown in Table 2 suggest that *C. luteum* suffers high mortality when compared to *D. quadrizonula* as the majority of its larvae or cocoons did not reach the adult stage.

### 3.3. Relative Abundance of Parasitoids

Table 3 shows the relative abundance of different FAW parasitoid species recorded in all districts in different seasons. The braconid *C. luteum* and the tachinid *D. quadrizonula* were the two most abundant species. While in the dry season the relative abundance of *C. luteum* oscillated from 3.7% in Vanduzi to 66.67% in Macate, in the rainy season, its relative abundance oscillated from 95% in Vanduzi to 100% in Macate, Manica, and Sussundenga. In the dry season, the abundance of *D. quadrizonula* varied from 25% in Macate to 96.3% in Vanduzi.

### 3.4. Parasitism Rates

Table 4 shows the parasitism rates of different parasitoid species of FAW. Parasitism rates varied both per district and season of sampling. Parasitism rates also varied among species with *C. luteum* reaching a maximum of 23.68% in the district of Macate during the rainy season, and *D. quadrizonula* reaching 8.86% in the district of Sussundenga during the dry season. The parasitism rates of *C. luteum* appeared to be higher during the rainy season in all districts when compared to the dry season.

### 3.5. Relative Contribution to Total Parasitism

The total parasitism of FAW larvae as the result of the individual contribution of different parasitoid species was estimated at 9.49%. The braconid *C. luteum* and the tachinid *D. quadrizonula* were the main contributors for the total parasitism with 5.12% and 4.02%, respectively (Table 5).

## 4. Discussion

Jourdie [37] experienced a serious problem with incomplete development of hymenopteran parasitoids emerging from fall armyworm larvae. However, a different scenario was reported by Agboyi [34] in which around 95% of *C. luteum* individuals completed their development. From Table 2, it can be observed that *C. luteum* individuals which emerged from FAW larvae collected during the dry season suffered higher mortality than those emerging from FAW larvae collected in the rainy season. Based only in this observation, we were unable to determine the possible cause for such behavior.

A survey conducted by Agboyi [34] in Ghana and Benin, found a complex of braconid, ichneumonid, and tachinid parasitoids including *D. quadrizonula*, *C. luteum*, and *Charops* sp. occurring in both countries and *M.* cf. *discolor* occurring only in Ghana. In East Africa—Ethiopia, Kenya, and Tanzania—*C. luteum* and *Charops* sp. were also found parasitizing FAW larvae with relatively high rates [32]. The braconid *C. luteum* is known to attack the following species: *Spodoptera exempta* Walker, *Spodoptera exigua* Hubner, *Condica capensis* Guenée all of them belonging to Lepidoptera: Noctuidae, and *Crypsotidia mesosema* Hampson (Lepidoptera: Erebidae) and *Cydia ptychora* (Meyrick) (Lepidoptera: Tortricidae). The ichneumonid *M.* cf. *discolor* is known to attack other species of Lepidoptera: Noctuidae namely: *Helicoverpa armigera* (Hubner), *Helicoverpa zea* Boddie, and *Spodoptera litura* Fabricius [38].

Braconid wasps seem to be good parasitoids for exhibiting high parasitism rates [24]. In this study, the braconid *C. luteum* was among the most common species and major contributors to the total parasitism. The importance of the endoparasitoid *C. luteum* as a biocontrol agent of FAW larvae in Africa was evidenced by Agboyi [39] when they observed a decrease in the leaf consumption in parasitized individuals by 89%.

Dipteran parasitoids are also reported as being important biocontrol agents of FAW in Argentina [40] and of *S. exempta,* a close relative of FAW in Nigeria [41]. Between the two recorded tachinid parasitoids, *D. quadrizonula* was the most common and also the major contributor to the total parasitism.

In our study, both ichneumonid *M.* cf. *discolor* and *Charops* sp. had low parasitism rates of 0.73% and 0.21%, respectively. Low parasitism rates of ichneumonid parasitoids on FAW larvae were also reported in Mexico [2,23] and Argentina [40]. However, in Tanzania, *Charops* sp. was found parasitizing up to 75% of the larvae of other lepidopteran pests such as *Spodoptera litoralis* Boisduval (Lepidoptera: Noctuidae) [42] and *Orgyia mixta* Snellen (Lepidoptera: Erebidae) [43], which are both close relatives of FAW. In another study, *M.* cf. *discolor* and *Charops* sp. were also reported as being parasitoids of *H. armigera*, another close relative of FAW [44].

Múrua [40] reported total parasitism of FAW by larval parasitoids as being around 35%. In our study, we recorded total parasitism of 9.49%, which is around four-fold lower. Pest species with similar characteristics as FAW namely, broad geographic distribution, wide host range, and high migratory behavior can easily escape, at least initially, from the constraints imposed by their native natural enemies [45]. This fact may explain low parasitism levels recorded in our study, given that FAW is a new pest in Mozambique. High parasitism rates of FAW in its native environment can be attributed to a large number of parasitoid species attacking targeting several stages, which was not the case observed in this study.

It is believed that biological control through habitat management may lead to a more sustainable pest control approach [46]. The fact that most smallholder farmers do not use insecticides in maize production should be considered as an advantage for the implementation of IPM programs based on biological control of FAW. Although it may take a considerable time to achieve a balanced relationship between FAW and its native parasitoids, the implementation of cultural practices favoring the action of parasitoids should be advocated.

## 5. Conclusions

The existence of a complex of native parasitoids of FAW in Mozambique, some of which are among the most common larval parasitoids of FAW such as the cases of *C. luteum* and *D. quadrizonula* can be used as the basis for the design of sustainable and farmer-friendly methods of control of FAW. The combined use of egg parasitoids of FAW such as *T. remus* which already occurs in Africa [33] and *Trichogramma* spp. with the already occurring larval parasitoids should be considered for a significant reduction of FAW population. For an optimization of the impact of these parasitoids, it is crucial to monitor and determine their role in the dynamics of FAW population as suggested by López [47]. It is also vital to avoid blanket application of insecticides, as per recommendation of Sisay [32]. Whether or not biological control of FAW will be adopted for smallholder farmers, it is important to note that pest management approaches should be flexible and respond to local situations as predetermined solutions may not work everywhere [48].

## Figures and Tables

**Table 1:** Distribution of fall armyworm (FAW) parasitoids per district and season of sampling.

Parasitoid Species	Host Stage Attacked	Behavior and Trait	Macate		Manica		Sussundenga		Vanduzi	
			DS	RS	DS	RS	DS	RS	DS	RS
*Coccygidium luteum*	Larva	Endoparasitoid and solitary	+	+	+	+	+	+	+	+
*Charops* sp.	Larva	Endoparasitoid and solitary	+	−	+	−	+	−	−	−
*Metopius* cf. *discolor*	Larva	***	−	−	−	−	+	−	−	−
Unidentified tachinid	Larva	***	−	−	+	−	−	−	−	−
*Drino quadrizonula*	Larva	Endoparasitoid and solitary-gregarious	+	−	−	−	+	−	+	+

*** The behavior and trait could not be determined because only one specimen was collected. DS = dry season; RS = rainy season; (−) = no record; (+) = present.

**Table 2:** Survival rates of different parasitoids emerging from FAW larvae per district and season of sampling.

Parasitoid Species	Macate		Manica		Sussundenga		Vanduzi	
	DS	RS	DS	RS	DS	RS	DS	RS
*Coccygidium luteum*	0 (n = 8)	44.44 (n = 9)	0 (n = 3)	52.63 (n = 19)	10 (n = 10)	20 (n = 5)	100 (n = 1)	31.58 (n = 19)
*Charops* sp.	100 (n = 1)	−	100 (n = 1)	−	100 (n = 1)	−	−	−
*Metopius* cf. *discolor*	−	−	−	−	100 (n = 1)	−	−	−
Unidentified tachinid	−	−	100 (n = 1)	−	−	−	−	−
*Drino quadrizonula*	100 (n = 3)	−	−	−	85.71 (n = 28)	−	88.46 (n = 26)	100 (n = 1)

DS = dry season; RS = rainy season; n= number of larvae of different parasitoid species emerging from FAW larvae.

**Table 3:** Relative abundance of FAW parasitoids per district and season of sampling.

Parasitoid Species	Macate		Manica		Sussundenga		Vanduzi	
	DS (n = 12)	RS (n = 9)	DS (n = 5)	RS (n = 19)	DS (n = 40)	RS (n = 5)	DS (n = 27)	RS (n = 20)
*Coccygidium luteum*	66.67	100	60.00	100	25.00	100	3.70	95.00
*Charops* sp.	8.33	−	20.00	−	2.50	−	−	−
*Metopius* cf. *discolor*	−	−	−	−	2.50	−	−	−
Unidentified tachinid	−	−	20.00	−	−	−	−	−
*Drino quadrizonula*	25.00	−	−	−	70.00	−	96.30	5.00

DS = dry season; RS = rainy season; n = total number of individuals of different parasitoid species.

**Table 4:** Parasitism rates of different FAW parasitoids per district and season of sampling.

Parasitoid Species	Macate		Manica		Sussundenga		Vanduzi	
	DS (n = 188)	RS (n = 38)	DS (n = 247)	RS (n = 115)	DS (n = 316)	RS (n = 63)	DS (n = 303)	RS (n = 174)
*Coccygidium luteum*	4.26	23.68	1.21	16.52	3.16	7.94	0.33	10.92
*Charops* sp.	0.53	−	0.40	−	0.32	−	−	−
*Metopius* cf. *discolor*	−	−	−	−	0.32	−	−	−
Unidentified tachinid	−	−	0.40	−	−	−	−	−
*Drino quadrizonula*	1.6	−	−	−	8.86	−	8.58	0.57

DS = dry season; RS = rainy season; n = number of FAW larvae collected.

**Table 5:** Relative contribution of different FAW parasitoids to total parasitism (N = 1444).

Parasitoid Species	Relative Parasitism
*Coccygidium luteum* (n = 74)	5.12
*Charops* sp. (n = 3)	0.21
*Metopius* cf. *discolor* (n = 1)	0.07
Unidentified tachinid (n = 1)	0.07
*Drino quadrizonula* (n = 58)	4.02
Total (n = 137)	9.49

n = number of FAW larvae parasitized by different parasitoid species; N = number of FAW larvae collected.

## Supplementary Materials

The following are available online at https://www.mdpi.com/2075-4450/11/9/615/s1, Table S1: Survival of individuals of different parasitoid species emerging from field-collected larvae.
