# Supplementary material for: First Report of Native Parasitoids of Fall Armyworm Spodoptera frugiperda Smith (Lepidoptera: Noctuidae) in Mozambique
Source: Insects. 2020 Sep 8;11(9):615. doi: 10.3390/insects11090615 (PMC7564170; doi:10.3390/insects11090615)
Supplement: Supplementary file 1 [file insects-11-00615-s001.pdf]

Table S1. Survival of parasitoid species.

Parasitoid species	Districts and season of sampling																							
	District of Macate						District of Manica						District of Sussundenga						District of Vanduzi					
	Dry Season			Rainy Season			Dry Season			Rainy Season			Dry Season			Rainy Season			Dry Season			Rainy Season		
	T	A	D	T	A	D	T	A	D	T	A	D	T	A	D	T	A	D	T	A	D	T	A	D
Coccygidium luteum (Brullé) (Hym.: Braconidae)	8	-	8	9	4	5	3	-	3	19	10	9	10	1	9	5	1	4	1	1	-	19	6	13
Charops sp. (Hym.: Ichneumonidae)	1	1	-	-	-	-	1	1	-	-	-	-	1	1	-	-	-	-	-	-	-	-	-	-
Metopius discolor Tosquinet? (Hym.: Ichneumonidae)	-	-	-	-	-	-	-	-	-	-	-	-	1	1	-	-	-	-	-	-	-	-	-	-
Unidentified (Dipt.: Tachinidae)	-	-	-	-	-	-	1	1	-	-	-	-	-	-	-	-	-	-	-	-	-	-	-	-
Drino quadrizonula (Thomson) (Dipt.: Tachinidae)	3	3	-	-	-	-	-	-	-	-	-	-	28	24	4	-	-	-	26	23	3	1	-	1

T: total number of individuals emerged from FAW larvae; A: number of individuals which reached adult stage; D: number of individuals which died at larval or pupal stage.
